# Ruthenium–Cyclopentadienyl–Cycloparaphenylene Complexes: Sizable Multicharged Cations Exhibiting High DNA-Binding Affinity and Remarkable Cytotoxicity

**DOI:** 10.3390/molecules29020514

**Published:** 2024-01-19

**Authors:** Konstantinos Ypsilantis, Evangelia Sifnaiou, Antonia Garypidou, Dimitrios Kordias, Angeliki Magklara, Achilleas Garoufis

**Affiliations:** 1 Laboratory of Inorganic Chemistry, Department of Chemistry, University of Ioannina, 45110 Ioannina, Greece; k_ipsilantis@yahoo.gr (K.Y.); e.sifnaiou@uoi.gr (E.S.); a.garypidou@uoi.gr (A.G.); 2 Biomedical Research Institute, Foundation for Research and Technology, 45110 Ioannina, Greece; d.kordias@hotmail.com (D.K.); magklara@uoi.gr (A.M.); 3 Laboratory of Clinical Chemistry, Faculty of Medicine, University of Ioannina, 45110 Ioannina, Greece; 4 Institute of Biosciences, University Research Center of Ioannina (U.R.C.I.), 45110 Ioannina, Greece; 5 Institute of Materials Science and Computing, University Research Centre of Ioannina (U.R.C.I.), 45110 Ioannina, Greece

**Keywords:** ruthenium, cycloparaphenylene, cyclopentadienyl, DNA binding

## Abstract

Two novel sizable multicharged cationic complexes, of the formulae [(*η*^6^–-[12]CPP)[Ru(*η*^5^–-Cp)]_12_]Χ_12_ and [(*η*^6^–-[11]CPP)[Ru(*η*^5^–-Cp)]_11_]Χ_11_, CPP = cycloparaphenylene, Cp = cyclopentadienyl, X = [PF_6_]^−^, (**1**), (**3**) and [Cl]^−^, (**2**), (**4**), were synthesized and characterized using NMR techniques, high-resolution mass spectrometry, and elemental analyses. Complexes (**1**) and (**3**) were stable in acetone and acetonitrile solutions over 48 h. In contrast, the water-soluble (**2**) and (**4**) begin to decompose in aqueous media after 1 h, due to the [Cl]^−^ tendency for nucleophilic attack on ruthenium of the {Ru(*η*^5^–-Cp)} units. Fluorescence quenching experiments conducted during the stability window of (**2**) with the d(5′-CGCGAATTCGCG-3′)_2_-EtBr adducts revealed remarkably high values for K_sv_ = 1.185 × 10^4^ ± 0.025 M^−1^ and K_b_ = 3.162 × 10^5^ ± 0.001 M^−1^. Furthermore, the cytotoxic activity of (**2**) against A2780, A2780res, and MCF-7 cancer cell lines shows that it is highly cytotoxic with IC_50_ values in the range of 4.76 ± 1.85 to 16 ± 0.81 μΜ.

## 1. Introduction

In recent years, cycloparaphenylenes have attracted significant attention from researchers due to their unique photophysical and photochemical properties [1,2]. The synthesis of both classic [3,4,5,6] and modified [7,8,9] cycloparaphenylenes, along with their potential applications, has opened a new frontier in chemistry. This field is not extensively explored yet, but it holds great promise for biomedical applications. In one instance, 10 [CPP] was observed to form vesicles, under specific conditions, that could cause some cytotoxicity after internalization by the cells. This example illustrates the potential use of these supramolecular structures as functional carbon biomaterials [10]. Jasti and colleagues synthesized a modified *m*-cycloparaphenylene serving as a fluorophore agent for biological imaging. This subcellular-targeted cycloparaphenylene, designed for both one- and two-photon live-cell imaging, exhibits no cytotoxicity up to 50 μM. Investigations into cellular uptake indicate internalization through endocytic pathways [11]. In a separate study, a water-soluble cycloparaphenylene was synthesized and functionalized with sulfonate groups. This water-soluble compound demonstrates minimal cytotoxicity and possesses the capability to enter the cellular environment, functioning as a bioimaging substance. Additionally, this compound exhibits insensitivity to pH changes, a crucial property given the varying pH values in different cellular parts [12].

Extensive evaluations of numerous transition metal complexes have revealed promising potential as anticancer agents [13,14,15,16]. Among them, ruthenium compounds have demonstrated remarkable cytotoxicity, showing significant promise for clinical applications. More particularly, ruthenium–arene organometallic compounds have attracted considerable research attention, primarily for two reasons: (a) their in vitro significant cytotoxicity in various cancer cell lines, and (b) their low cytotoxicity in healthy cells. The latter presents a critical advantage compared to platinum anticancer complexes. The exploration of the cytotoxic activity of ruthenium–arene compounds started with the study of the half-sandwich complex [(*η*^6^–C_6_H_6_)Ru(metronidazole)Cl_2_] (where metronidazole is 1-β-hydroxyethyl-2-methyl-5-nitroimidazole) [17]. A few years later, Saddler’s group reported that the complex [(*η*^6^–cym)Ru(en)Cl]PF_6_ exhibits antitumor activity similar to carboplatin across a wide range of cancer cell lines [18]. This finding triggered a series of investigations into half-sandwich ruthenium–arene complexes with various chelate ligands. Among these complexes, those containing aromatic diamines instead of ethylenediamine have exhibited remarkable cytotoxicity and demonstrated a capacity for binding to DNA, as has been reported by our group [19,20,21,22].

However, the lack of selectivity towards cancer cells and the irreversible coordinative binding of ruthenium–arene half-sandwich complexes to targeted biomolecules prompted the design of complexes with kinetically inert ligands. These complexes are able to bind non-coordinatively and reversibly to biomolecules, potentially demonstrating anticancer activity. A category of compounds displaying these characteristics is the class of full-sandwich ruthenium–arene complexes. More specifically, cationic full-sandwich complexes containing *η*^5^–cyclopentadienyl units have shown anticancer activity, albeit through a different mechanism than that of the half-sandwich arene complexes. The absence of binding due to the lack of leaving groups results in a different mechanism of action [23,24]. It has been reported that {Ru(*η*^5^–Cp*)} complexes with various *η*^6^–arene ligands show remarkable cytotoxicity, depending on the size and the lipophilicity of the arene ligand [25]. Bulkier and more lipophilic ligands tend to increase the complexes’ cytotoxicity. Kudinov et al. discovered that complexes [(*η*^5^–L1)Ru(L2)]BF_4_ comprising an amino acid of the type [(C_5_Me_4_CH_2_OOCCH(CH_2_R)NHBoc)Ru(C_10_H_8_)]BF_4_ (R = tryptophan, phenylalanine) showed notable cytotoxicity in various cancer cell lines with IC_50_ = 21–96 μΜ, which is similar to that of cisplatin [26]. The same group explored the selective labeling of the tryptophan residue in melittin, an existing peptide in bee venom with potential anticancer activity, using the organometallic fragment {RuCp} [27]. The substantial stability of the [(*η*^5^–C_5_H_5_)Ru(*η*^6^–Trp-melittin)] allowed for establishing the biodistribution of the modified protein in mice through inductively coupled plasma MS analysis of ruthenium [28]. It also has been reported that the bimetallic complex [(*η*^5^–Cp*)Ru(*η*^6^–benz-*η*^5^–Cp)Fe(*η*^5^–Cp)]Cl and the trinuclear bimetallic one [(*η*^5^-Cp*)Ru(*η*^6^-benz-*η*^5^-Cp)Fe(*η*^5^-Cp-*η*^6^-benz)Ru(*η*^5^-Cp*)]Cl exhibited significant cytotoxic activity against various cancer cell lines (A2780, SK-OV-3, MDA-MB-231) [29]. Given this significant cytotoxicity observed in polynuclear full-sandwich ruthenium–arene complexes, we considered that cycloparaphenylenes may be ideal ligands.

To date, only a limited number of metal complexes with modified or unmodified cycloparaphenylenes have been reported, yet none of these have been screened for cytotoxic activity. Itami’s group synthesized a symmetrically modified cycloparaphenylene incorporating two 2,2′-bipyridine units, named cyclo [14] paraphenylene[4]2,5-pyridylidene ([14,4]CPPy). The resulting bimetallic complex of [14,4]CPPy with Pd(II) is a promising starting material for applications in synthesizing supramolecular nanotubes [30]. In a related study by Jasti’s group, they embedded a single unit of 2,2′-bipyridine into the smaller cycloparaphenylene [8]CPP [31]. The corresponding complexes [Ru(bpy)_2_(2,2′–bipy[8]CPP)](PF_6_)_2_ and Pd(2,2′–bipy[8]CPP)Cl_2_ and Pd(2,2′–bipy[8]CPP)_2_](BF_4_)_2_ synthesized with good yields and exhibited unique solid-state photophysical properties. Also, the modification of [9]CPP via embedding a 2,2′-bipyridine unit afforded the modified cycloparaphenylene, bpy[9]CPP, which was further reacted with Fe(H_2_B(pyz)_2_)_2_Cl_2_, forming the complex [Fe(bipy–[9]CPP){H_2_B(pyz)_2_}_2_]. Magnetic susceptibility measurements suggest spin-crossover behavior with a T_1/2_ = 130 K [32]. The substantial number of phenylene units in unmodified cycloparaphenylene, along with their chemical equivalence, poses challenges for *η*^6^-coordination by several metals. Initially, Itami et al. successfully synthesized a series of complexes through the *η*^6^-coordination of [9]CPP or [12]CPP with M(CO)_6_ (M = Cr, Mo, W) with the general formula [(*η*^6^–-[n]CPP)M(CO)_3_], (n = 9, 12; M = Cr, Mo, W). It was observed that the complex [(*η*^6^–-[9]CPP)Cr(CO)_3_] leads to selective monolithiation only for the *η*^6^-coordinated phenylene unit [33]. Additionally, Yamago facilitated a regioselective synthesis of small cycloparaphenylenes (5[CPP] and 6[CPP]) *η*^6^-coordinated with various number of {Ru(η^5^-Cp)} units [34].

In our previous work, we observed that the complexes of the general formula [(*η*^6^–-[12]CPP)[Ru(*η*^5^–-Cp)]_n_]Cl_n_ (n = 2–10) release solvolyzed {RuCp} species upon UV irradiation. In the presence of 9-methylguanine (9-MeG) and subsequent irradiation with UV, adducts between {RuCp} and 9-MeG were observed [35]. To further investigate the biological properties of the Ru(*η*^5^-Cp)-cycloparaphenylene multinuclear complexes, we hereby report on the synthesis and characterization of the sizable multicharged cationic complexes [(*η*^6^–-[12]CPP)[Ru(*η*^5^–-Cp)]_12_](PF_6_)_12_ and [(*η*^6^–-[11]CPP)[Ru(*η*^5^–-Cp)]_11_](PF_6_)_11_. The DNA binding properties and the cytotoxic activity against various cancer cell lines of the [(*η*^6^–-[12]CPP)[Ru(*η*^5^–-Cp)]_12_]Cl_12_ were also investigated.

## 2. Results and Discussion

### 2.1. Synthesis and Characterization of [(η^6^–-[12]CPP)[Ru(η^5^–-Cp)]_12_](PF_6_)_12_ and [(η^6^–-[11]CPP)[Ru(η^5^–-Cp)]_11_](PF_6_)_11_

**Scheme 1:**
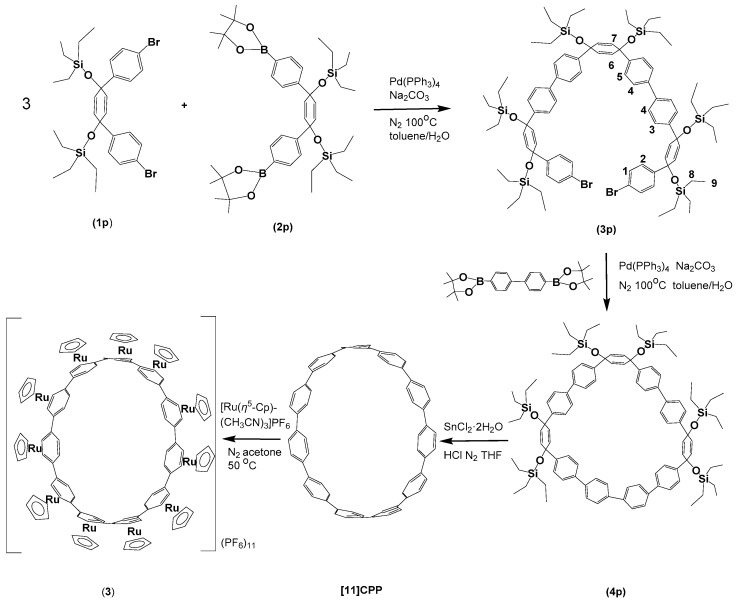
Synthetic procedure for the synthesis of (**3**), and structure numbering of (**3p**).

Recently, S. Yamago et al. [34] synthesized complexes of [6]CPP and [5]CPP, *η*^6^–-coordinated with {Ru(*η*^5^–-Cp)} units. The choice of solvent and the differing solubilities of the complexes resulted in the isolation of the mono- and binuclear complexes [(*η*^6^–-[5]CPP)Ru(*η*^5^–-Cp)](PF_6_) and [(*η*^6^–-[5]CPP)[Ru(*η*^5^–-Cp)]_2_](PF_6_)_2_, respectively. The [6]CPP produced a trinuclear complex *η*^6^-coordinated with three {Ru(*η*^5^-Cp)} units in alternate sites to each paraphenylene [34]. Building on this concept, in our previous work, we synthesized complexes of the type [(*η*^6^–-[12]CPP)[Ru(*η*^5^–-Cp)]_n_]Cl_n_ (n = 2–10) which, however, was isolated as mixtures of various n. Following the same approach, we synthesized and isolated the complexes [(*η*^6^–-[12]CPP)[Ru(*η*^5^–-Cp)]_12_](PF_6_)_12_ and [(*η*^6^–-[11]CPP)[Ru(*η*^5^–-Cp)]_11_](PF_6_)_11_. The complete *η*^6^-coordination of [11]CPP and [12]CPP with {Ru(*η*^5^–-Cp)} units demands more time, higher temperatures, and an excess of [(*η*^5^–-Cp)Ru(CH_3_CN)_3_]PF_6_. The gradual addition of the [(*η*^5^–-Cp)Ru(CH_3_CN)_3_] and the significant excess in the second dosage, assisted by heating, leads to fully coordinated complexes. The choice of the acetone as solvent is a crucial factor, since the intermediates coordinated with various numbers of {Ru(*η*^5^–-Cp)} must remain soluble. The complexes were precipitated after the addition of CH_2_Cl_2_ to the reaction mixture and washed many times with CH_2_Cl_2_ to remove the various unreacted {RuCp} species before being analyzed using mass spectrometry and NMR spectroscopy. The synthetic procedure for (**3**) summarized in Scheme 1.

**Figure 1:**
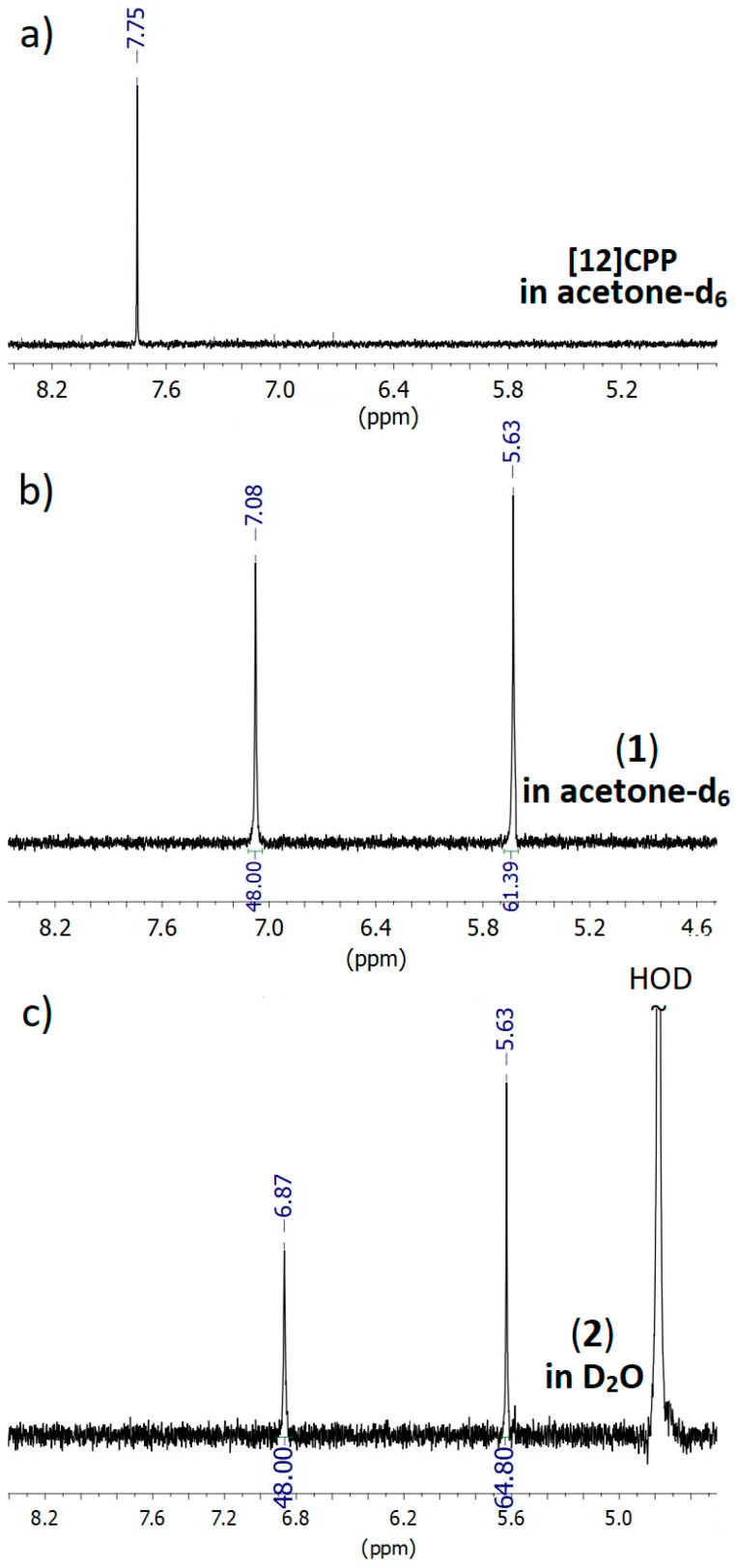
^1^H NMR spectra of [12]CPP in acetone-d_6_ (**a**), (**1**) in acetone-d_6_ (**b**), and (**2**) in D_2_O (**c**).

The ^1^H NMR spectrum of (**1**) showed two singlet peaks. The signal at 7.08 ppm was assigned to *η*^6^–-coordinated phenyl rings with the {RuCp} units, shifted upfield by −0.54 ppm compared to the free [12]CPP. This shift reflects the contribution of ruthenium to the arene electron density [34,36]. On the other hand, the cyclopentadienyl protons appeared as a single singlet peak at 5.63 ppm, indicating a symmetrical distribution of the Cp rings around the [12]CPP. Similarly, a ^1^H NMR spectrum was observed in the case of (**3**) (Figure S1). The corresponding [Cl]^−^ salts, (**2**) and (**4**), showed similar spectra, but the observed signals were significantly upfield, likely due to hydrophobic intramolecular interactions in D_2_O (Figure 1).

**Figure 2:**
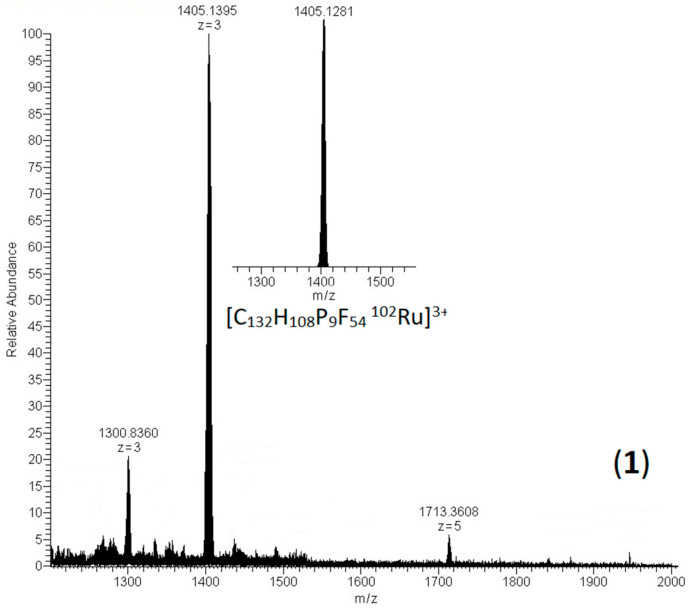
The positive mode of the high-resolution ESI mass spectrum of (**1**) showing a main cluster-peak at *m*/*z* = 1405.1395. Inset the calculated cluster-peak for the triple-charged cation [C_132_H_108_P_9_F_54_^102^Ru_12_]^3+^.

**Figure 3:**
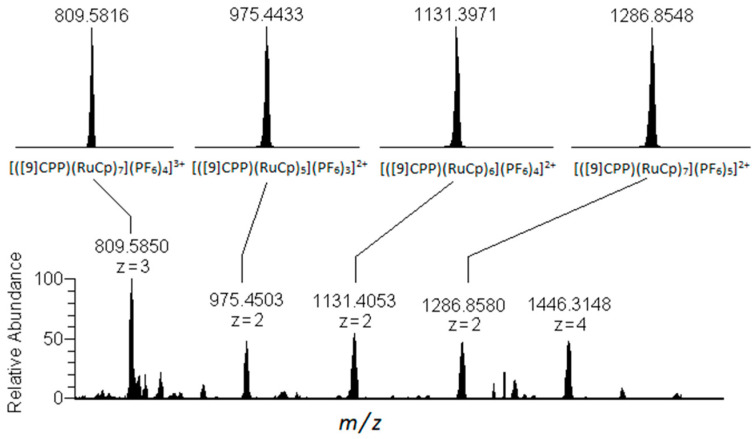
The positive mode of the high-resolution ESI mass spectrum of the reaction mixture, containing [9]CPP and an excess of [(*η*^5^–-Cp)Ru(CH_3_CN)_3_]PF_6_, shows the formation of various species of the complexes [(*η*^6^–-[9]CPP)[Ru(*η*^5^–-Cp)]_n_](PF_6_)_n_, with n ranging from 5 to 7.

The HR-ESI mass spectra of the complexes (**1**) (Figure 2) and (**3**) (Figure S2) showed a triple-charged cluster-peak assigned to the cation {[(*η*^6^–-[12]CPP)[Ru(*η*^5^–-Cp)]_9_](PF_6_)_9_}^3+^, and two cluster-peaks, one triple-charged and one quarter-charged, assigned to the cations {[(*η*^6^–-[11]CPP)[Ru(*η*^5^–-Cp)]_8_](PF_6_)_8_}^3+^ and {[(*η*^6^–-[11]CPP)[Ru(*η*^5^–-Cp)]_7_](PF_6_)_7_}^4+^, respectively. However, the spectra of (**2**) and (**4**) consisted of many different species of the formulae [(*η*^6^–-[12 or 11]CPP)[Ru(*η*^5^–-Cp)]_n_](Cl)_n_ with n = 5–10. This reflects the relatively lower stability of (**2**) and (**4**) [Cl]^−^ salts, in contrast to the more stable [PF_6_]^−^. The explanation for the above behavior may lie in the fact that the anion [Cl]^−^ is capable of nucleophilic attack on the {Ru(*η*^5^-Cp)} units, destabilizing complexes (**2**) and (**4**), while the [PF_6_]^−^ is unable to coordinate, maintaining the stability of (**1**) and (**3**).

We have made several unsuccessful attempts to isolate fully *η*^6^-coordinated complexes of smaller cycloparaphenylenes such as [9]CCP and [6]CPP with {Ru(*η*^5^–-Cp)}. In the case of [9]CPP, we attempted the in-situ formation of the complex [(*η*^6^–-[9]CPP)[Ru(*η*^5^–-Cp)]_9_](PF_6_)_9_ via adding a high excess of [(*η*^5^–-Cp)Ru(CH_3_CN)_3_]PF_6_. However, the NMR and mass spectra analyses revealed species attributed only to [(*η*^6^–-[9]CPP)[Ru(*η*^5^–-Cp)]_n_](PF_6_)_n_ with n = 5–7, (Figure 3). Similarly, efforts to synthesize the complex [(*η*^6^-[6]CPP)[Ru(*η*^5^–-Cp)]_6_](PF_6_)_6_ in situ by monitoring the ^1^H NMR spectrum of the reaction mixture showed species corresponding to the complex [(η^6^–-[6]CPP)[Ru(*η*^5^–-Cp)]_3_](PF_6_)_3_, but not the fully coordinated one, with six {Ru(*η*^5^–-Cp)} units. However, it appears that this regioselectivity of the {RuCp} moieties on the nanoring is absent in larger cycloparaphenylenes (n > 10). Upon investigating the cycloparaphenylene [5]CPP, it was observed that only two {Ru(η^5^–-Cp)} units could coordinate, which are less than half of the potential units for coordination. In the case of 6[CPP], the coordination with {Ru(*η*^5^–-Cp)} occurred just for three units, indicating that the coordination of neighboring phenylene units leads to a less thermodynamically stable complex [34]. Regarding larger CPPs, the number of coordination sites seems to be random, forming in addition a small amount of the fully coordinated complex.

The synthesis and stability of compounds (**1**) and (**3**) suggest that the critical factor for the *η*^6^-coordination of the {Ru(*η*^5^–-Cp)} units lies in the convexity of the CPP acquired by the phenyl ring. In smaller CPPs, the nonplanar phenyl rings adopt a shallow boat conformation due to the ring strain, resulting in a dihedral angle of the out-of-plane carbon atom calculated as 15.6° for [5]CPP [37]. This conformation seems to hinder the *η*^6^-coordination of the {Ru(*η*^5^–-Cp)} unit. However, when ruthenium coordinates, it reduces this angle, causing an increase in the corresponding angle of the neighboring phenyl rings to maintain the ring shape. Consequently, only three {Ru(*η*^5^–-Cp)} units can coordinate alternately to [6]CPP [34]. In larger CPPs, this angle is smaller [37], reducing the impact on the neighboring phenyl rings upon {Ru(*η*^5^-Cp)} coordination, facilitating the continuous coordination of the units.

Although it is assumed that the {Ru(*η*^5^–-Cp)} moiety tends to favor the concave side of a curved aromatic organic molecule [38,39] due to its higher negative electrostatic potential compared to the convex surface [40], its ability to coordinate concavely with smaller CPPs is impeded due to steric reasons. However, in larger CPPs where steric hindrance is insignificant due to the size of CPP, the deviation from the planarity in the phenyl rings becomes marginal. Consequently, the {Ru(*η*^5^–-Cp)} moiety coordinates from the exterior side of the nanoring.

### 2.2. Stability Studies of the Complex in Aqueous Media

Although initially isolated as [PF_6_]^−^ salts, complexes (**1**) and (**3**) were converted into their [Cl]^−^ salts, which are soluble in aqueous media, in order to study their cytotoxic properties. Both complexes (**1**) and (**3**) are stable in acetone and acetonitrile for over 48 h. In contrast, their [Cl]^−^ salts, (**2**) and (**4**), are stable in aqueous media only for a short period, releasing solvolyzed {Ru(*η*^5^–-Cp)} species. Monitoring the ^1^H NMR spectrum of a freshly prepared solution of (**2**), we observed a new peak at about 7.75 ppm, attributed to the proton signals of an unbound phenyl ring. Essentially, this indicates that a {Ru(*η*^5^–-Cp)} unit has dissociated from (**2**), leaving a phenyl ring unbound. In parallel, a signal at 6.5 ppm appeared, attributed to cyclopentadienyl protons of various {RuCp} solvolyzed species.

In fact, the integrals ratio of these new signals is 4:5, precisely reflecting the ratio between the protons of an unbound phenyl group in the [12]CPP and the ligand cyclopentadienyl, indicating their association originated from the same release reaction. Over time, these signals broadened, and additional peaks appeared due to the formation of various RuCp species on one side, and phenyl ring protons located in random sites on (**2**) on the other side. Both the solvent and the [Cl]^−^ seem to contribute to the decomposition of (**2**), participating in the formation of various {Ru(*η*^5^-Cp)} species.

**Figure 4:**
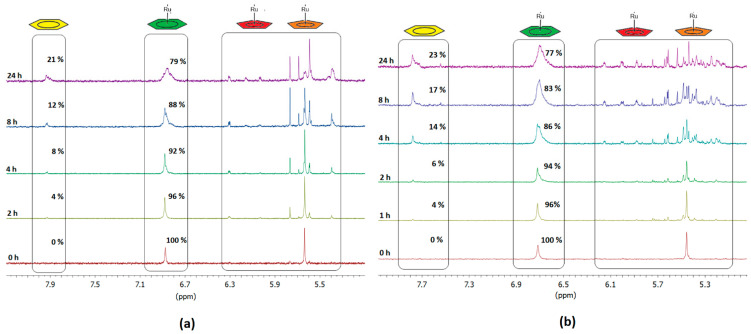
Aromatic region of ^1^H NMR spectra at 298 K showing the progressive release of ruthenium species for (**2**), (**a**), and (**4**), (**b**). (Note: After 24 h, there are no significant changes observed).

Integrating the four-proton signals of the unbound phenyl rings of (**2**), appearing around 7.9 ppm (Figure 2, yellow ring) and comparing them with those of the remaining [(*η*^6^–-[12]CPP)[Ru(*η*^5^–-Cp)]_n_](PF_6_)_n_, n < 12, (Figure 2, green ring), enables the determination of the decomposition percentage. Also, we can confirm the obtained results by integrating the five-proton signals of all the cyclopentadienyls, nanoringcoordinated, and released, appearing between 5.1–6.1 ppm (Figure 2 red and orange rings). However, the decomposition rate of (**2**) and (**4**) differs. Complex (**4**) exhibits a faster release of {Ru(*η*^5^–-Cp)} species, indicating a higher decomposition rate. This observation further supports the hypothesis that the curvature of a CPP, which is related to its size, significantly influences the coordination and the release of the {Ru(*η*^5^–-Cp)} units. After 24 h, both complexes have lost a similar percentage of {Ru(*η*^5^-Cp)} units: 21% for (**2**) and 23% for (**4**), suggesting a similar equilibrium. Figure 4 presents the decomposition (**2**) and (**4**) over time.

### 2.3. Biological Studies

#### 2.3.1. Fluorescence Quenching Studies of the d(5′-CGCGAATTCGCG-3′)_2_-Ethidium Bromide Adducts, with (**2**)

**Figure 5:**
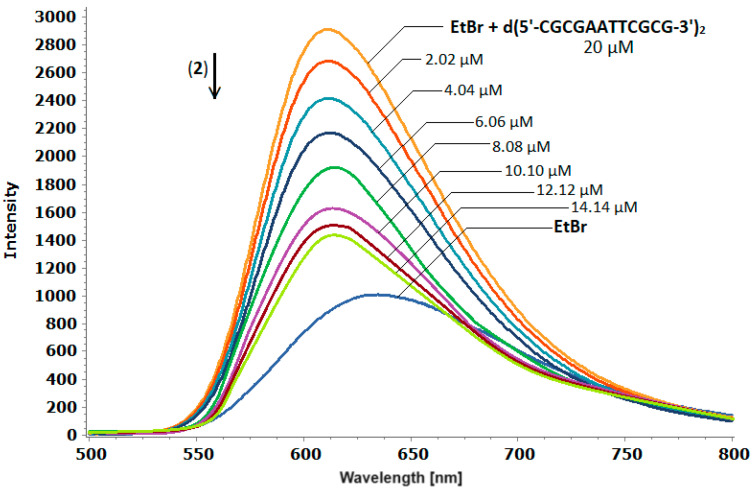
Fluorescence emission spectra of d(5′-CGCGAATTCGCG-3′)_2_-EtBr, titrated with (**2**) at 298 K. [DNA] = 20μM, [EtBr] = 5.2 μM and (**2**) from 0 to 12.40 μM.

Fluorescence spectroscopy stands out as a highly sensitive and effective method for investigating the binding affinity of small molecules with DNA [41]. Ethidium bromide (EtBr) exhibits three different binding modes with DNA: (i) primarily through intercalation, which significantly enhances DNA fluorescence [42,43]; (ii) within the helix minor groove; and (iii) via electrostatic interactions due to its cationic nature [44]. The displacement of EtBr from DNA-EtBr adducts via a DNA binder results in a notable quenching in emission intensity. The degree of the quenching directly correlates with the competitive binding constant (K_sv_). Molecular simulation studies investigating the interaction between the dodecamer d(5′-CGCGAATTCGCG-3′)_2_ and EtBr indicate its predominant stacking between the terminal base pairs CG of the sequence, with minimal interaction with the inner AT base pairs [45]. Investigating the affinity of (**2**) with the d(5′-CGCGAATTCGCG-3′)_2_-EtBr adducts, we prepared a solution d(5′-CGCGAATTCGCG-3′)_2_ in buffer phosphates (100 mM, pH = 7.0) to which we gradually added an amount of EtBr until the emission intensity reached the saturation point where any further addition has a negligible impact. Subsequently, a sample of the above stock solution was titrated with complex (**2**). It was observed that the emission intensity of the d(5′-CGCGAATTCGCG-3′)_2_-EtBr decreased rapidly as the concentration of (**2**) increased, while the λ of the emission maximum remained almost unchanged (Figure 5). This observation strongly suggests the displacement of the EtBr from the DNA-EtBr adduct due to its the interaction with (**2**). This outcome was unexpected, considering that (**2**) is capable of participating only in electrostatic interactions with the negatively charged phosphate groups of the d(5′-CGCGAATTCGCG-3′)_2_-EtBr.

On the other hand, it is known that cations with high charge density exhibit a considerable impact on the persistence length of the DNA [46]. Several methods have demonstrated significant bending of B-DNA induced by multivalent cations [47,48,49], even though some random sequence DNA maintains in B conformation [50,51]. Rouzina and Bloomfield proposed a mechanism by which multivalent cations induce DNA bending [46]. According to their model, a multivalent cation binds to the B-DNA major groove electrostatically, repelling the sodium counterions from neighboring phosphates. This results in a strong binding of the cation to the groove, accompanied by groove closure and DNA bending toward the captured cation. In the context of the electrostatic interaction between the dodecavalent cation [(*η*^6^–-[12]CPP)[Ru(*η*^5^–-Cp)]_12_]^12+^ and the d(5′-CGCGAATTCGCG-3′)_2_-EtBr, it is possible that significant bending of the helix occurs, causing the release of EtBr and subsequent quenching of the emission intensity of the sample. This release occurs not through the replacement of EtBr by another molecule, but simply due to structural alterations of the B-DNA conformation induced by the strong electrostatic interaction. The above results reflected in the competition Stern–Volmer quenching constant, the value of which was determined from the slope of the F/F_o_ = *f*([Q]) plot (Figure S3) as K_sv_ = 1.185 × 10^4^ ± 0.025 M^−1^, indicating a great displacement of the EtBr from the duplex d(5′-CGCGAATTCGCG-3′)_2_. The calculated binding constant K_b_, obtained through the double logarithmic plot log[(F_0_–-F)/F] versus log[Q] (Figure S4), is 3.162 × 10^5^ ± 0.001 M^−1^. This value indicates a strong affinity of (**2**) with the d(5′-CGCGAATTCGCG-3′)_2_, similar to those of the DNA intercalators [52], signifying more than a simple external electrostatic interaction.

#### 2.3.2. Cytotoxic Activity

**Figure 6:**
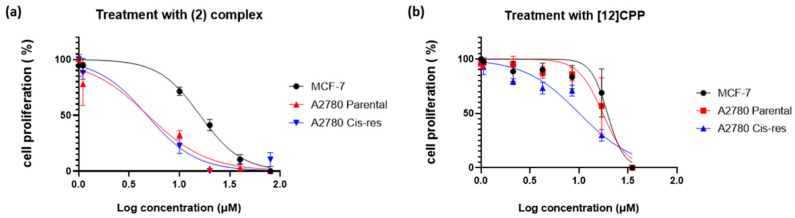
Effects of (**2**) and [12]CPP complexes on cell growth. The human breast cancer cell line MCF-7 and the human ovarian cancer cell lines A2780 and A2780 Cis-res were treated for 72 h with 1–80 μM and 1–35 μM of the (**2**) (**a**) and the [12]CPP (**b**) complexes, respectively. Cell growth rate was determined through confluency measurements using the Incucyte Zoom live cell analysis system. Data from two to three independent biological experiments performed in triplicate are shown. Error bars represent the S.E. of the mean.

To study the effects of (**2**) and [12]CPP on cell growth in vitro, three human cancer cell lines were treated with different concentrations of these compounds for 72 h and their IC_50_ values were calculated. The cytotoxic effects of the two compounds were monitored by employing real-time imaging using the Incucyte ZOOM system and the results are shown in Figure 6. Complex (**2**) exhibited the most potent effect on the proliferation of the ovarian cancer cell line A2780 and its cisplatin-resistant counterpart (A2780 Cis-res) (Figure 6a) depicting similar IC_50_ values (4.85 and 4.76 μΜ, respectively) (Table 1). It is interesting that both cell lines were equally sensitive to complex (**2**), suggesting that this compound has the potential to be used as a second-line treatment after the development of cisplatin resistance in ovarian cancer. Complex (**2**) showed a weaker, but still potent, cytotoxic effect on the breast adenocarcinoma cell line MCF-7 (Figure 6a) with the IC_50_ value being 16 μM (Table 1). The A2780 Cis-res cells were quite susceptible to the cytotoxic effect of the [12]CPP compared to the other two cell lines (Figure 6b). Specifically, the IC_50_ value of [12]CPP in the resistant cells was ~10 μM, while it was ~17.5 μΜ and ~19.5 μΜ in the A2780 parental and in the MCF-7 cells, respectively (Table 1). Overall, complex (**2**) was more efficient than the [12]CPP one, especially in the ovarian cancer cells.

## 3. Experimental

### 3.1. Materials and Methods

Tris(acetonitrile)cyclopentadienylruthenium(II) hexafluorophosphate, [(*η*^5^–-Cp)Ru(CH_3_CN)_3_]PF_6_ was purchased from Ruthenotope. [12]CPP, [9]CPP and [6]CPP, were synthesized according to literature [35,53,54]. (((1′s,4′s)-4,4″-dibromo-1′,4′-dihydro-[1,1′:4′,1″-terphenyl]-1′,4′-diyl)bis(oxy))bis(triethylsilane) (**1p**) and (((1′s,4′s)-4,4″-bis(4,4,5,5-tetramethyl-1,3,2-dioxaborolan-2-yl)-1′,4′-dihydro-[1,1′:4′,1″-terphenyl]-1′,4′-diyl)bis (oxy))bis(triethylsilane) (**2p**) were synthesized according to published methods [7]. 4,4′-bis(4,4,5,5-tetramethyl-1,3,2-dioxaborolan-2-yl)-1,1’-biphenyl, was purchased from TCI Chemicals. The rest of the reagents we used were purchased from Alfa Aesar and TCI Chemicals. The deoxynucleotide d(5′-CGCGAATTCGCG-3′) was purchased from Eurogentec. All solvents were of analytical grade and used after proper purification. The deuterated solvents, acetone-d_6_, D_2_O, CDCl_3_ and CD_2_Cl_2_, were purchased from Sigma and were of >99.9% purity. C, H, and N determinations were performed on a PerkinElmer 2400 Series II analyzer. ^1^H NMR spectra were recorded on Bruker Avance spectrometers operating in ^1^H frequencies of 400.13 and 500.13 MHz and processed using Topspin 2.1 (Bruker Analytik GmbH, Billerica, MA, USA). High resolution electrospray ionization mass spectra (HR-ESI-MS) of the complexes were obtained on an Agilent Technology LC/MSD trap SL instrument and Thermo Scientific LTQ Orbitrap XL™ system (Waltham, MA, USA).

### 3.2. Preparation of Ligands and Complexes

(**3p**): Into a 50 mL reaction vial tube equipped with a small magnetic stir bar, we added 100 mg (0.135 mmol, 1 eq.) (**1p**), 262 mg (0.405 mmol, 3 eq.) (**2p**), 15.4 mg (1.014 mmol 0.1 eq.) tetrakis(triphenylphosphine)palladium(0), and 141 mg (1.350 mmol, 10 eq.) Na_2_CO_3_. The vial was then sealed with a screw cap and purged with dry nitrogen for 1 h. Subsequently, a mixture of 2 mL water and 16 mL of toluene underwent freeze−pump−thaw (3 times) before being added to the reaction vial. The sealed vial was then placed in a 100 °C oil bath and stirred vigorously for 16 h. After cooling the reaction mixture at room temperature, the crude product was extracted with DCM/H_2_O, concentrated to about 1 mL, and purified by column chromatography (SiO_2_, 10/90, ethyl acetate/hexane) affording (**3p**). Yield 50%. Anal. for C_90_H_126_Br_2_O_6_Si_6_, cal.: C: 66.22%; H: 7.78%, found: C: 66.33%; H: 7.64%. HR-ESI-MS, positive (*m*/*z*): 1630.6641, calc. 1630.6511 for [C_90_H_126_Br_2_O_6_Si_6_]^+^. ^1^H NMR (500 MHz, 298 K, CD_2_Cl_2_, *δ* in ppm): H1, 7.55 (d, 4H); H2, 7.55 (d, 4H); H3, 7.27 (d, 4H); H4, 7.41 (d, 8H); H5, 7.47 (d, 4H); H6, 6.09 (d, 6H); H7, 6.02 (d, 4H); H8, 0.67 (m, 36H); H9, 0.98 (m, 54H) (see Figures S5a and S6).

[11]CPP: The [11]CPP was synthesized with a slight modification to the published method [3]. Into a 10 mL reaction vial tube equipped with a small magnetic stir bar, we added 108 mg (0.066 mmol, 1 eq.) of (**3p**), 32mg (0.073 mmol, 1.2 eq.) of 4,4’-bis(4,4,5,5-tetramethyl-1,3,2-dioxaborolan-2-yl)-1,1’-biphenyl, 7.7 mg (0.006 mmol, 0.1 eq.) of tetrakis(triphenylphosphine)-palladium(0), and 69.6 mg (0.662 mmol, 10 eq.) of Na_2_CO_3_. The vial was then sealed with a screw cap and purged with dry nitrogen for 1 h. Subsequently, a mixture of 2 mL water and 16 mL of toluene underwent freeze−pump−thaw (3 times) before being added to the reaction vial. The sealed vial was then placed in a 100 °C oil bath and vigorously stirred for 16 h. After cooling the reaction mixture at room temperature, we extracted the product with DCM/H_2_O, evaporated to about 1 mL and passed through column chromatography of SiO_2_ with 15/85 ethyl acetate/hexane as eluent. The main product of the column (**4p**) was evaporated to dryness and proceeded without further purification.

To a THF (5 mL) solution containing 89.8 mg (0.40 mmol) SnCl_2_·2H_2_O, we added 0.1 mL HCl 12 M (1.20 mmol) at room temperature and stirred for 0.5 h. The resulting solution was then added to another 5 mL solution of THF containing 0.05 mL conc. HCl and 110 mg of (**4p**). After stirring for 12 h at 50 °C we added 5 mL of an aqueous solution NaOH (10% *w*/*v*) and performed extraction with DCM. The residue obtained was purified via column chromatography (SiO_2_, 1/2, CH_3_Cl/hexane) to give [11]CPP as a pale yellow solid. Yield 3,2%. The ^1^H NMR spectrum was consistent with the literature data in CDCl_3_ [3] and it was recorded in acetone-d_6_ (Figure S7).

[(*η*^6^–-[12]CPP)[Ru(*η*^5^–-Cp)]_12_](PF_6_)_12_, (**1**): We dissolved 5 mg of [12]CPP (0.005 mmol, 1 eq.) in 4 mL of dry acetone and to this solution we added 28.5 mg (0.06 mmol, 12 eq.) of [(*η*^5^-Cp)Ru(CH_3_CN)_3_]PF_6_. The reaction mixture was kept under nitrogen atmosphere at 50 °C for 6 h. After this duration, an additional amount of 14.3 mg (0.06 mmol, 6 eq.) [(*η*^5^-Cp)Ru(CH_3_CN)_3_]PF_6_ was added, and the mixture was left to react at 50 °C for another 18 h. Evaporation of the solvents to 0.5 mL was carried out and 10 mL of DCM was added. Immediately, a brown precipitate was obtained, which was washed with DCM (5 × 2 mL) and diethyl ether (2 × 3 mL) and dried under vacuum. The brown solid was stable at −20 °C for a long period of time. Yield 35%. Anal. for C_132_H_108_F_72_P_12_Ru_12_ (calc.): C: 34.12%; H: 2.34%; (found): C: 34.22%; H: 2.29%. HR-ESI-MS, positive (*m*/*z*): found 1405.1395, calc. 1405.1281 for [C_132_H_108_P_9_F_54_^102^Ru_12_]^3+^. ^1^H NMR (400 MHz, 298 K, acetone-d_6_, *δ* in ppm): phH, 7.08 (s, 48H); CpH, 5.63 (s, 60H).

[(*η*^6^–-[12]CPP)[Ru(*η*^5^–-Cp)]_12_]Cl_12_, (**2**): The [PF_6_]^−^ anion of (**1**) was replaced by [Cl]^−^ as described earlier [40a]. It was stable at −20 °C for 30 days. Yield 95%. Anal. for C_132_H_108_Ru_12_Cl_12_, (cal.): C: 47.57%; H: 3.27%; (found): C: 47.52%; H: 3.29%. ^1^H NMR (400 MHz, 298 K, D_2_O, *δ* in ppm): phH, 6.87 (s, 48H); CpH, 5.63 (s, 60H).

[(*η*^6^–-[11]CPP)[Ru(*η*^5^–-Cp)]_11_](PF_6_)_11_, (**3**): The complex (**3**) was synthesized in a similar manner to (**1**) Anal. for C_121_H_99_F_66_P_11_Ru_11_, (calc.): C: 34.12%; H: 2.34%; (found): C: 34.07%; H: 2.45%; HR-ESI-MS, positive (*m*/*z*): found 920.6272, calc. 920.6202 for [C_121_H_99_P_7_F_42_^102^Ru_11_]^4+^, found 1275.8116, calc. 1275.8151 for [C_121_H_99_P_8_F_48_^102^Ru_11_]^3+^. ^1^H NMR (400 MHz, 298 K, acetone-d_6_, *δ* in ppm): phH, 7.07 (s, 44H); CpH, 5.61 (s, 55H).

[(*η*^6^–-[11]CPP)[Ru(*η*^5^–-Cp)]_11_](Cl)_11_, (**4**): The [PF_6_]^−^ anion of complexes (**3**) was replaced by [Cl]^−^ as described earlier [40a]. It was stable at −20 °C for a period of 30 days. Yield 98%. Anal. for C_121_H_99_Ru_11_Cl_11_, (cal.): C: 47.57%; H: 3,27%; (found): C: 47.55%; H: 3.25%. ^1^H NMR (400 MHz, 298 K, D_2_O, *δ* in ppm): phH, 6.72 (s, 44H); CpH, 5.45 (s, 55H).

### 3.3. Stability Studies of the Complex in Aqueous Media

The stability of the [Cl]^−^ and [PF_6_]^−^ salts of the complexes in organic solvents and aqueous media was monitored by ^1^H NMR (Figures S8 and S9) and HR-ESI-MS (Figures S10 and S11) spectroscopy. In a typical experiment, 1–2 mg of each complex was dissolved in 0.5 mL of the appropriate solvent in order to obtain a 2 mM solution and the ^1^H NMR spectrum of the sample was recorded at intervals of 15 min for the initial 5-h period.

### 3.4. Fluorescence Measurements

Fluorescence emission study was carried out using a Jasco FP-8300 fluorimeter equipped with a xenon lamp source. All the experiments were performed using a 10 mm path length cuvette in a 100 mM phosphate buffer at pH 7.0. Successive amounts of complex (**2**) from a stock solution of 1 mM were added to a 20 μM of d(5’-CGCGAATTCGCG-3’)_2_ saturated with ethidium bromide EtBr (5.07 μM) [55]. The DNA–EtBr sample was titrated with (**2**) and the emission spectra were recorded at wavelengths of 500–800 nm with excitation at 480 nm in a 1 cm quartz cell. The excitation and emission slit widths were kept at 5 nm each. All measurements were recorded following a 5 min incubation at 298 K to minimize the exposure of (**2**) to the aqueous environment and ensure that it remains intact (see stability studies). Details on the calculations of K_sv_ and K_b_ are presented in the Supplementary Materials.

### 3.5. Cell Culture

The human breast adenocarcinoma cell line MCF-7 was cultured in Dulbecco’s modified Eagle’s medium (DMEM, Sigma Cat. N. D6429, St. Louis, MI, USA) supplemented with 10% fetal bovine (Gibco, Cat. N. 10270-106, Waltham, MA, USA) serum and 1% penicillin/streptomycin (Biowest, Cat. N. L0022, Nuaillé, Maine-et-Loire,). The human ovarian cancer cell line A2780 and its cisplatin resistant derivative (A2780 Cis-res) were cultured in RPMI1640 medium (RPMI-1640, Sigma, Cat. N. R8758) supplemented with 10% fetal bovine serum (Gibco, Cat. N. 10270-106) and 1% penicillin/streptomycin (Biowest, Cat. N. L0022). Cells were routinely passaged every 2 or 3 days, and were incubated in a humidified atmosphere of 5% CO_2_ at 37 °C. A stock solution of 12[CPP] was prepared by dissolving a quantity of the solid in doubly distilled H_2_O containing 1% DMSO [10]. Also, the stock solution of complex (**2**) was prepared by dissolving the complex in doubly distilled H_2_O and immediately adding appropriate portions to the cell culture.

### 3.6. Cell Growth Assay

To monitor the cell growth and evaluate the cytoxicity effects of (**2**) and [12]CPP, the IncuCyte Zoom system (Essen BioScience, Hertfordshire, UK) and software were used, as has been previously described [20]. The IC_50_ values of the compounds were calculated from a log(concentration) versus normalized response curve fit using Graphpad Prism version 8.01. Two to three independent, biological experiments were conducted in triplicate for each compound in each cell line.

## 4. Conclusions

In summary, the synthesis, isolation, and characterization of the multinuclear, multicharged cationic complexes [(*η*^6^–-[12]CPP)[Ru(*η*^5^–-Cp)]_12_]^12+^ and [(*η*^6^–-[11]CPP)[Ru(*η*^5^–-Cp)]_11_]^11+^ resulted in the formation of their [PF_6_]^−^, (**1**) and (**3**), and [Cl]^−^, (**2**) and (**4**), salts. It was observed that the [Cl]^−^ anion in the cases of (**2**) and (**4**) exhibited a remarkable ability to make a nucleophilic attack on the coordinated {Ru(*η*^5^–-Cp)} units, destabilizing these complexes. In contrast, the [PF_6_]^−^ did not engage in coordination, thereby preserving the stability of complexes (**1**) and (**3**). Moreover, this study concluded that larger cycloparaphenylenes, such [11]CPP and [12]CPP, favor the continuous *η*^6^-coordination of {Ru(*η*^5^–-Cp)} units due to the reduced deviation from the planarity in their phenyl rings. This structural characteristic facilitates more stable complexes.

The sizable dodecacharged cation [(*η*^6^–-[12]CPP)[Ru(*η*^5^–-Cp)]_12_]^12+^ participates in electrostatic interactions with the EtBr-d(5’-CGCGAATTCGCG-3’)_2_ adducts, inducing substantial bending of the B-type helix conformation and consequent displacement of the EtBr. The calculated K_sv_ value of 1.185 × 10^4^ ± 0.025 M^−1^ indicates an almost complete displacement of EtBr from the duplex d(5′-CGCGAATTCGCG-3′)_2_. Additionally, the binding constant K_b_, calculated as 3.162 × 10^5^ ± 0.001 M^−1^, suggests a high affinity between (**2**) and the d(5′-CGCGAATTCGCG-3′)2, similar to the reported values for DNA intercalators [52]. This highlights a binding mode that surpasses a typical external electrostatic interaction. The cytotoxic activity of (**2**) against the A2780, A2780res, and MCF-7 cell lines reveals it is highly cytotoxic, making it much more effective than free [12]CPP.

## Figures and Tables

**Table 1:** IC_50_ values of compounds (**2**) and [12]CPP in the cancer cell lines MCF-7, A2780 and A2780 Cis-res. (Cisplatin IC_50_ against: MCF-7 = 11.88 ± 1.20, A2780 = 3.87 ± 0.33, A2780 Cis-res = 17.10 ± 0.24).

Compounds	MCF-7	A2780	A2780 Cis-Res
(**2**)	16 ± 0.81	4.85 ± 1.28	4.76 ± 1.85
[12]CPP	19.49 ± 2.67	17.46 ± 1.77	10.07 ± 1.09

## Data Availability

Data are contained within the article and Supplementary Materials.

## Supplementary Materials

The following supporting information can be downloaded at: https://www.mdpi.com/article/10.3390/molecules29020514/s1, Figure S1. ^1^H NMR spectrum of complex [(*η*^6^–-[11]CPP)[Ru(*η*^5^–-Cp)]_11_](PF_6_)_11_ (**3**) in acetone-d_6_. Figure S2. HR-ESI-MS spectrum of complex [(*η*^6^–-[11]CPP)[Ru(*η*^5^–-Cp)]_11_](PF_6_)_11_ (**3**). Figure S3. Stern–Volmer plots for the interaction of [(*η*^6^–-[12]CPP)[Ru(*η*^5^–-Cp)]_12_]Cl_12_ with d(5′-CGCGAATTCGCG-3′)_2_-EtBr at 298 K. Figure S4. The double-log plots of [(*η*^6^-[12]CPP)[Ru(*η*^5^–-Cp)]_12_]Cl_12_ fluorescence quenching effect, on d(5′-CGCGAATTCGCG-3′)_2_-EtBr at 298 K. Figure S5. ^1^H NMR spectrum of **3p** in CD_2_Cl_2_. Figure S6. HR-ESI-MS spectrum of **3p**. Figure S7. ^1^H NMR spectrum of [11]CPP in acetone-d_6_. Figure S8. ^1^H NMR spectrum of (**2**) in D_2_O after 48 h and 72 h. Figure S9. ^1^H NMR spectrum of (**4**) in D_2_O after 48 h and 72 h. Figure S10. HR-ESI-MS spectrum of (**2**). Figure S11. HR-ESI-MS spectrum of (**4**).
