# Supplementary material for: Ruthenium–Cyclopentadienyl–Cycloparaphenylene Complexes: Sizable Multicharged Cations Exhibiting High DNA-Binding Affinity and Remarkable Cytotoxicity
Source: Molecules. 2024 Jan 19;29(2):514. doi: 10.3390/molecules29020514 (PMC10818589; doi:10.3390/molecules29020514)
Supplement: Supplementary file 1 [file molecules-29-00514-s001.zip › molecules-2809969-supplementary.pdf]

Supplementary Material

Ruthenium-Cyclopentadienyl-Cycloparaphenylene Complexes: Sizable Multicharged Cations Exhibiting High DNA-Binding Affinity and Remarkable Cytotoxicity

Konstantinos Ypsilantis[1], Evangelia Sifnaiou[1], Antonia Garypidou[1], Dimitris Kordias[3,4], Angeliki Magklara[3,4,5], Achilleas Garoufis[1,2]

[1] Laboratory of Inorganic Chemistry, Department of Chemistry, University of Ioannina, 45110 Ioannina, Greece

[2] University Research Centre of Ioannina (URCI), Institute of Materials Science and Computing, 45110 Ioannina, Greece.

[3] Biomedical Research Institute-Foundation for Research and Technology, 45110 Ioannina, Greece

[4] Laboratory of Clinical Chemistry, Faculty of Medicine, University of Ioannina, 45110 Ioannina, Greece

[5] Institute of Biosciences, University Research Center of Ioannina (U.R.C.I.), 45110 Ioannina, Greece

Table of contents

FIGURE S1. ^{1}H NMR spectrum of complex $[(\eta^6\text{-[11]CPP})[Ru(\eta^5\text{-Cp})]_{11}](PF_6)_{11}$ (**3**) in acetone-d_6.
FIGURE S2. HR-ESI-MS spectrum of complex $[(\eta^6\text{-[11]CPP})[Ru(\eta^5\text{-Cp})]_{11}](PF_6)_{11}$ (**3**).
FIGURE S3. Stern–Volmer plots for the interaction of $[(\eta^6\text{-[12]CPP})[Ru(\eta^5\text{-Cp})]_{12}]Cl_{12}$ with d(5'-CGCGAATTCGCG-3')$_2$-EtBr at 298 K.
FIGURE S4. The double-log plots of $[(\eta^6\text{-[12]CPP})[Ru(\eta^5\text{-Cp})]_{12}]Cl_{12}$ fluorescence quenching effect, on d(5'-CGCGAATTCGCG-3')$_2$-EtBr at 298 K.
FIGURE S5. ^{1}H NMR spectrum of **3p** in CD_2Cl_2.
FIGURE S6. HR-ESI-MS spectrum of **3p**.
FIGURE S7. ^{1}H NMR spectrum of [11]CPP in acetone-d_6.
FIGURE S7. ^{1}H NMR spectrum of [11]CPP in acetone-d_6.
FIGURE S8. ^{1}H NMR spectrum of (**2**) in D_2O after 48h and 72h.
FIGURE S9. ^{1}H NMR spectrum of (**4**) in D_2O after 48h and 72h.
FIGURE S10. HR-ESI-MS spectrum of (**2**).
FIGURE S11. HR-ESI-MS spectrum of (**4**).

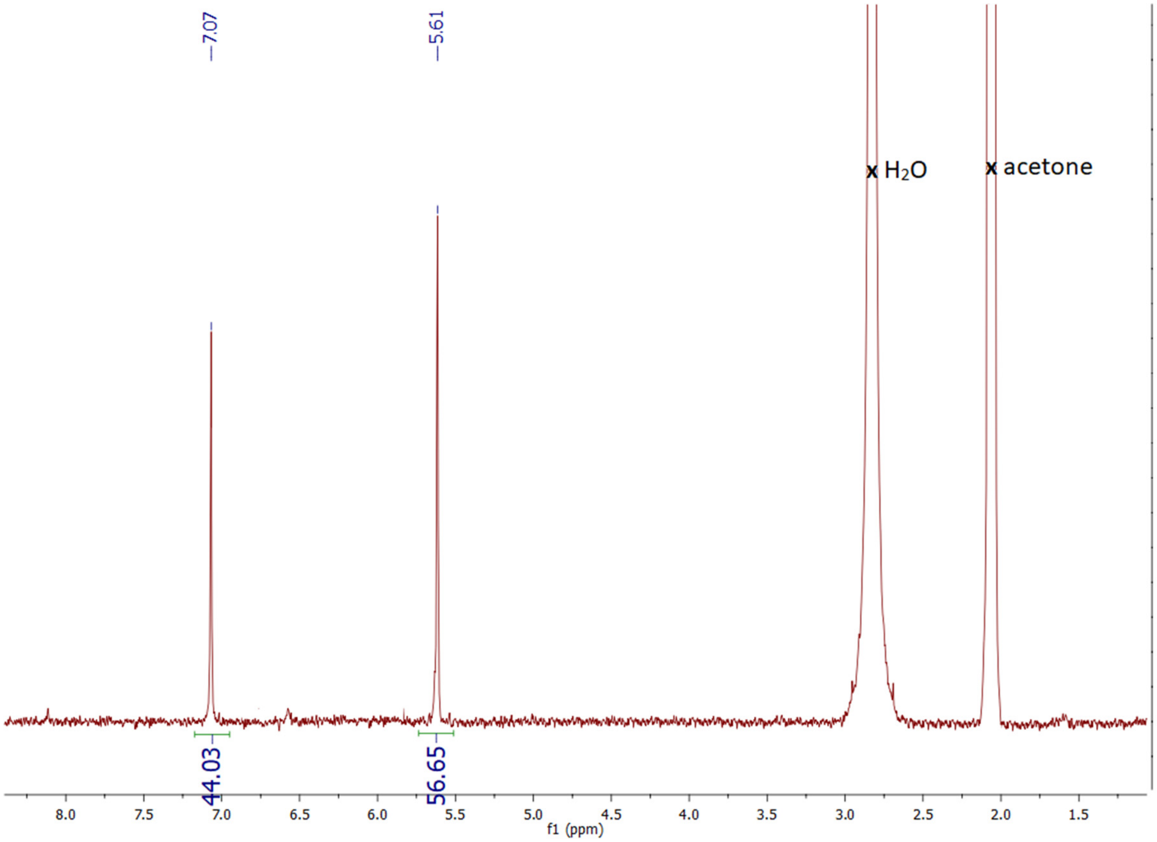

FIGURE S1. ^{1}H NMR spectrum of complex [(η^{6}-[11]CPP)[Ru(η^{5}-Cp)]$_{11}$](PF$_6$)$_{11}$ (**3**) in acetone-d$_6$.

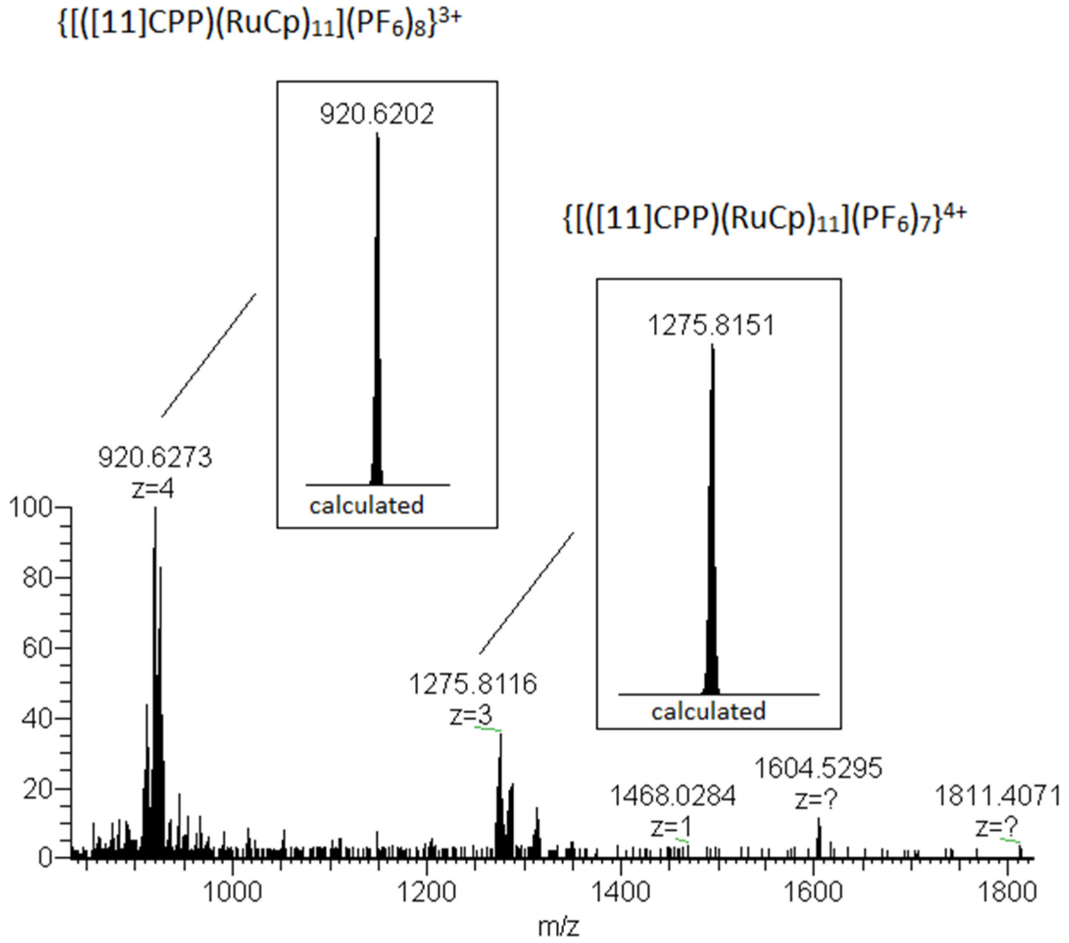

FIGURE S2. HR-ESI-MS spectrum of complex $[(\eta^6\text{-}[11]\text{CPP})[\text{Ru}(\eta^5\text{-Cp})]_{11}](\text{PF}_6)_{11}$ (**3**)

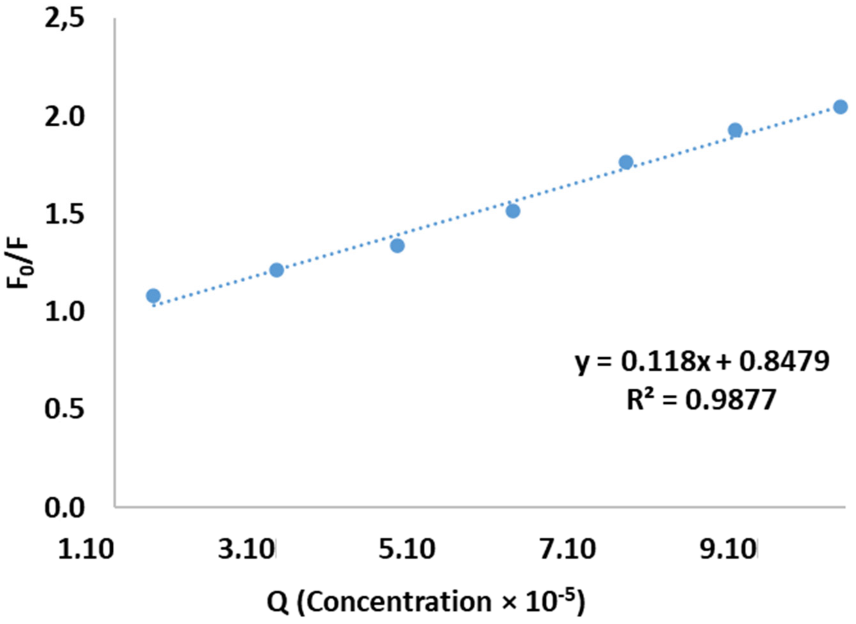

FIGURE S3. Stern–Volmer plots for the interaction of [(η^6-[12]CPP)[Ru(η^5-Cp)]$_{12}$]Cl$_{12}$ with d(5'-CGCGAATTCGCG-3')$_2$-EtBr at 298 K

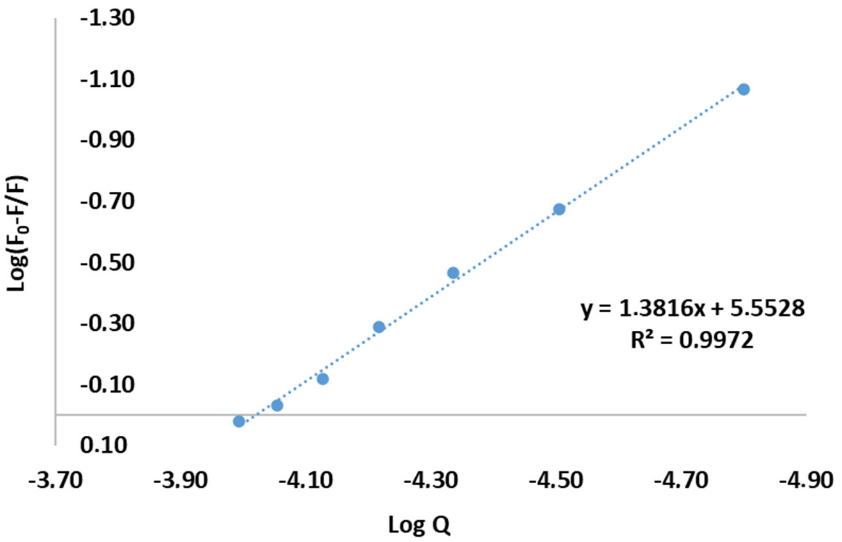

FIGURE S4. The double-log plots of [(η^6-[12]CPP)[Ru(η^5-Cp)]$_{12}$]Cl$_{12}$ fluorescence quenching effect, on d(5'-CGCGAATTCGCG-3')$_2$-EtBr at 298 K.

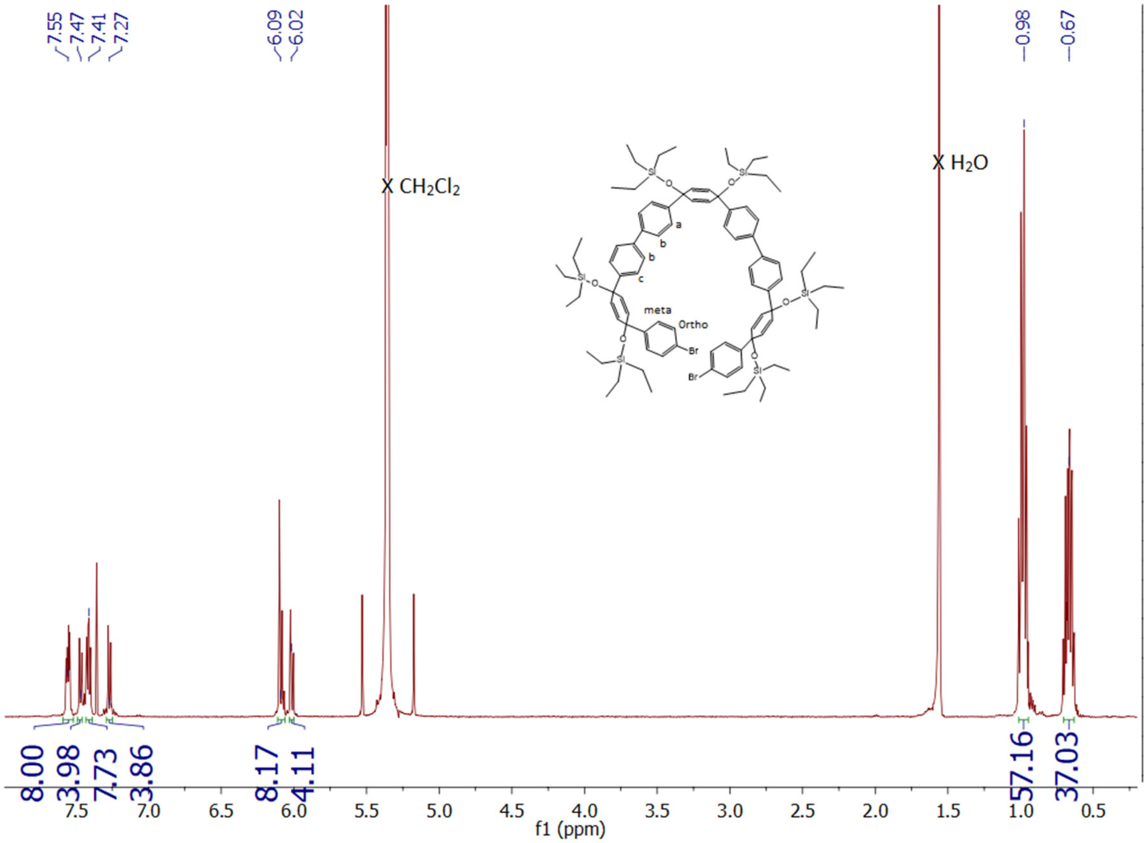

FIGURE S5. ^{1}H NMR spectrum of **3p** in CD_2Cl_2.

3p

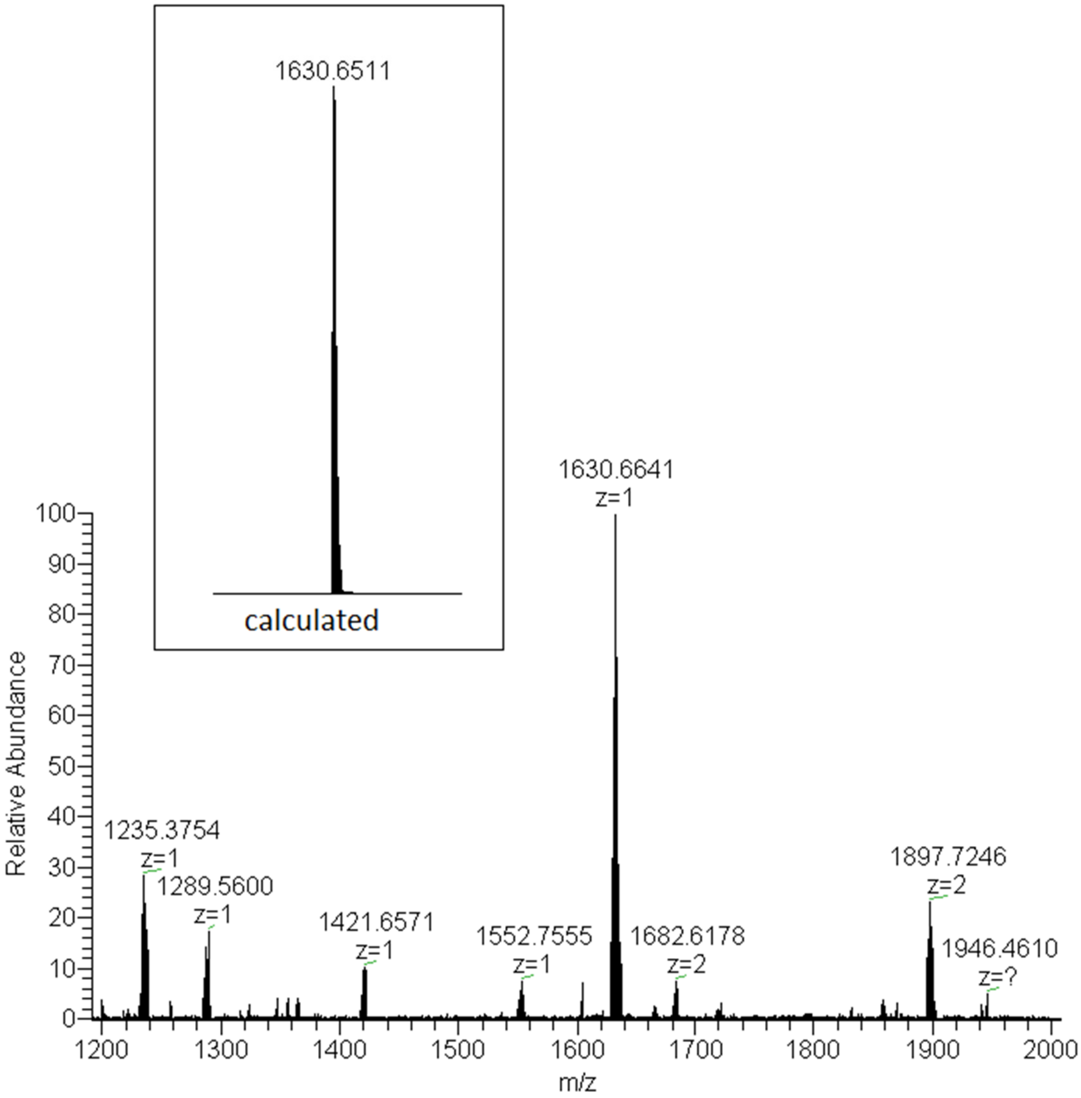

FIGURE S6. HR-ESI-MS spectrum of **3p**.

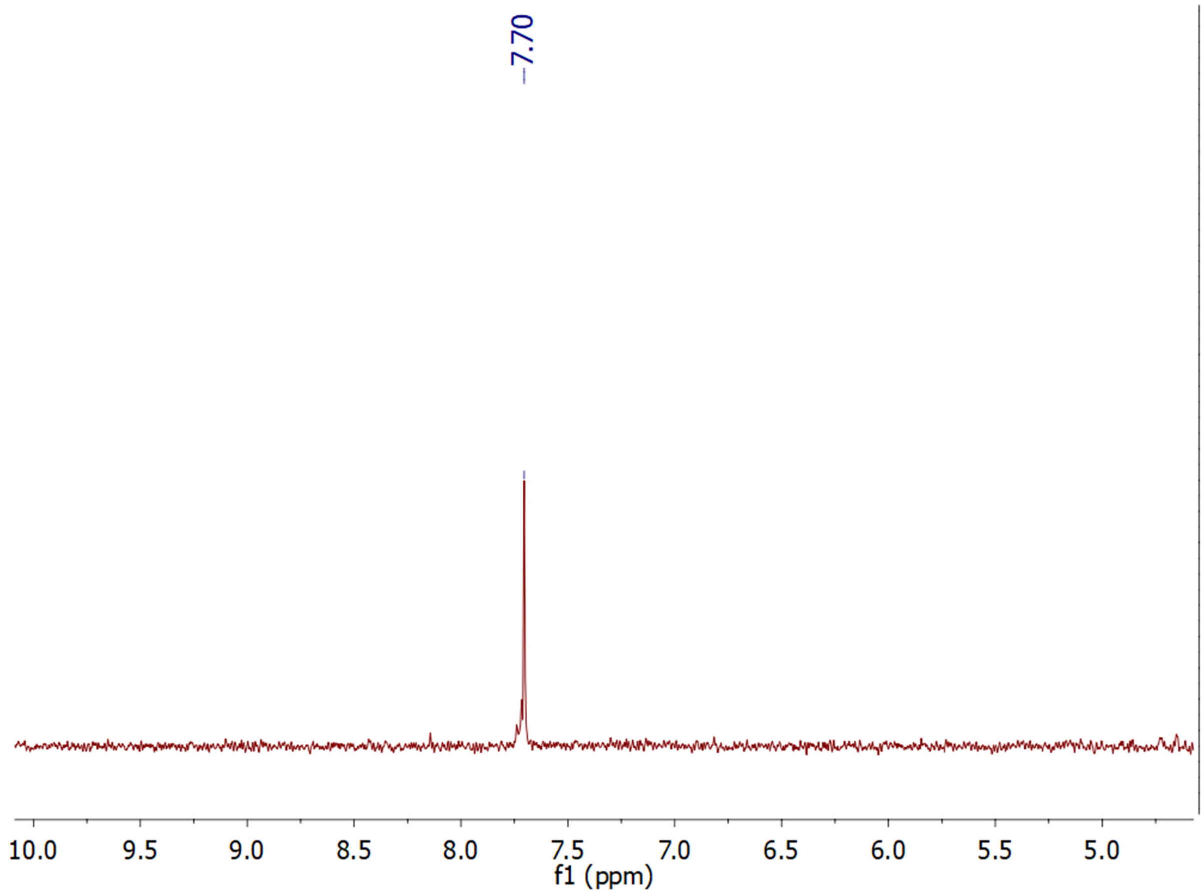

FIGURE S7. ^{1}H NMR spectrum of [11]CPP in acetone-d_6.

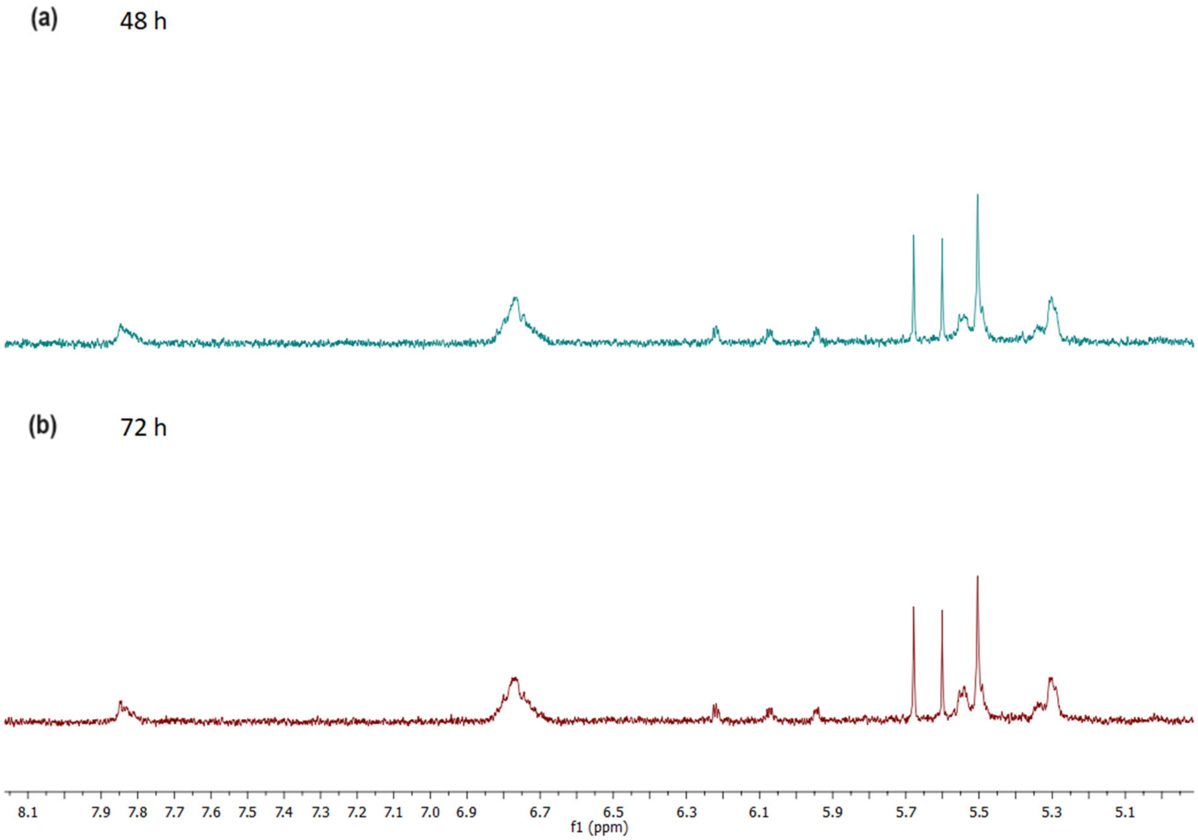

FIGURE S8. ^1H NMR spectrum of (**2**) in D_2O after 48h and 72h.

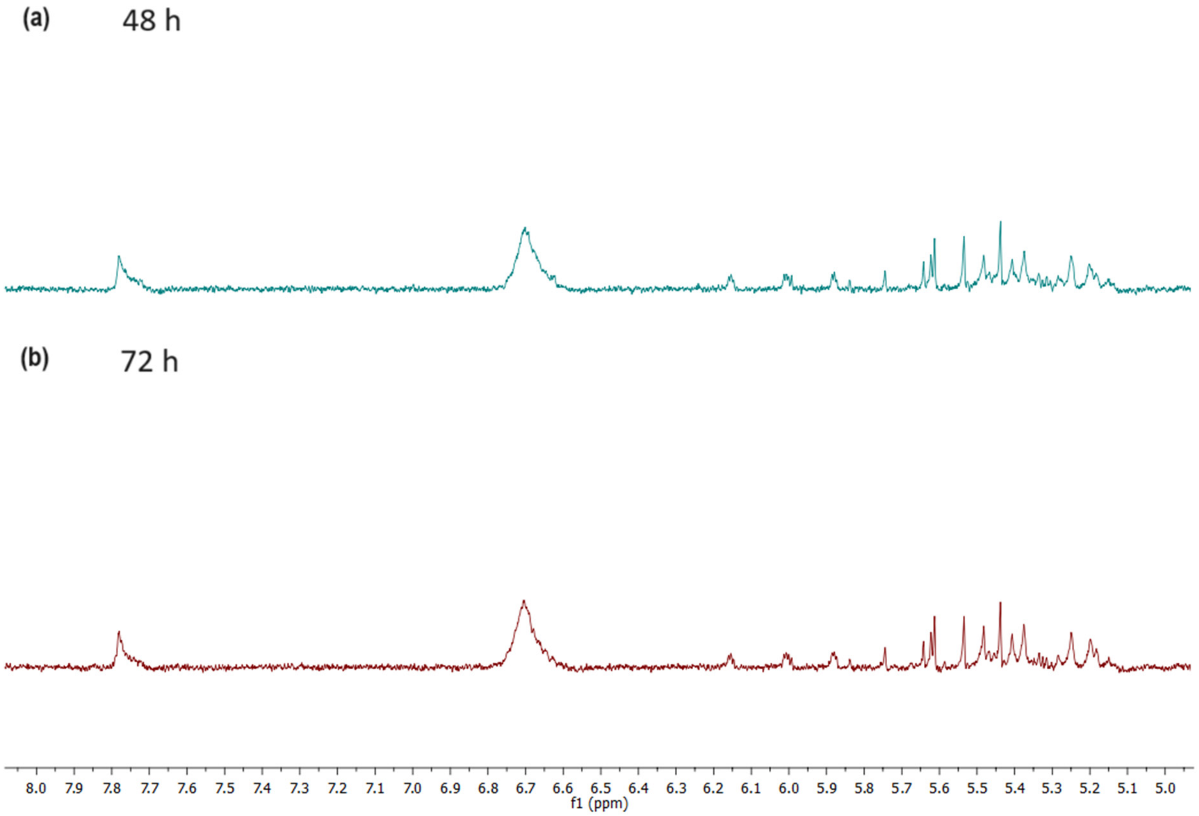

FIGURE S9. ^{1}H NMR spectrum of (**4**) in D_2O after 48h and 72h.

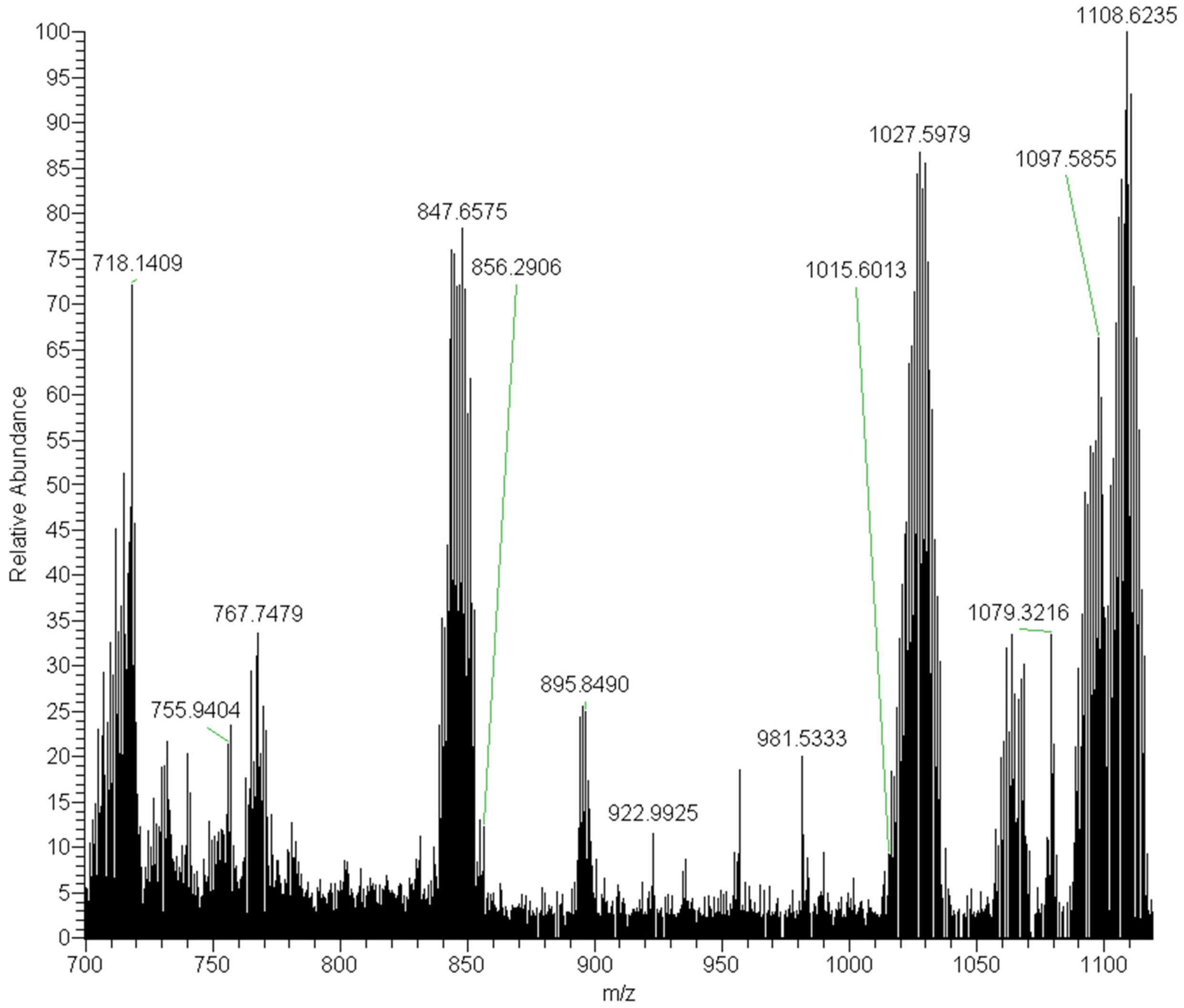

FIGURE S10. HR-ESI-MS spectrum of (**2**).

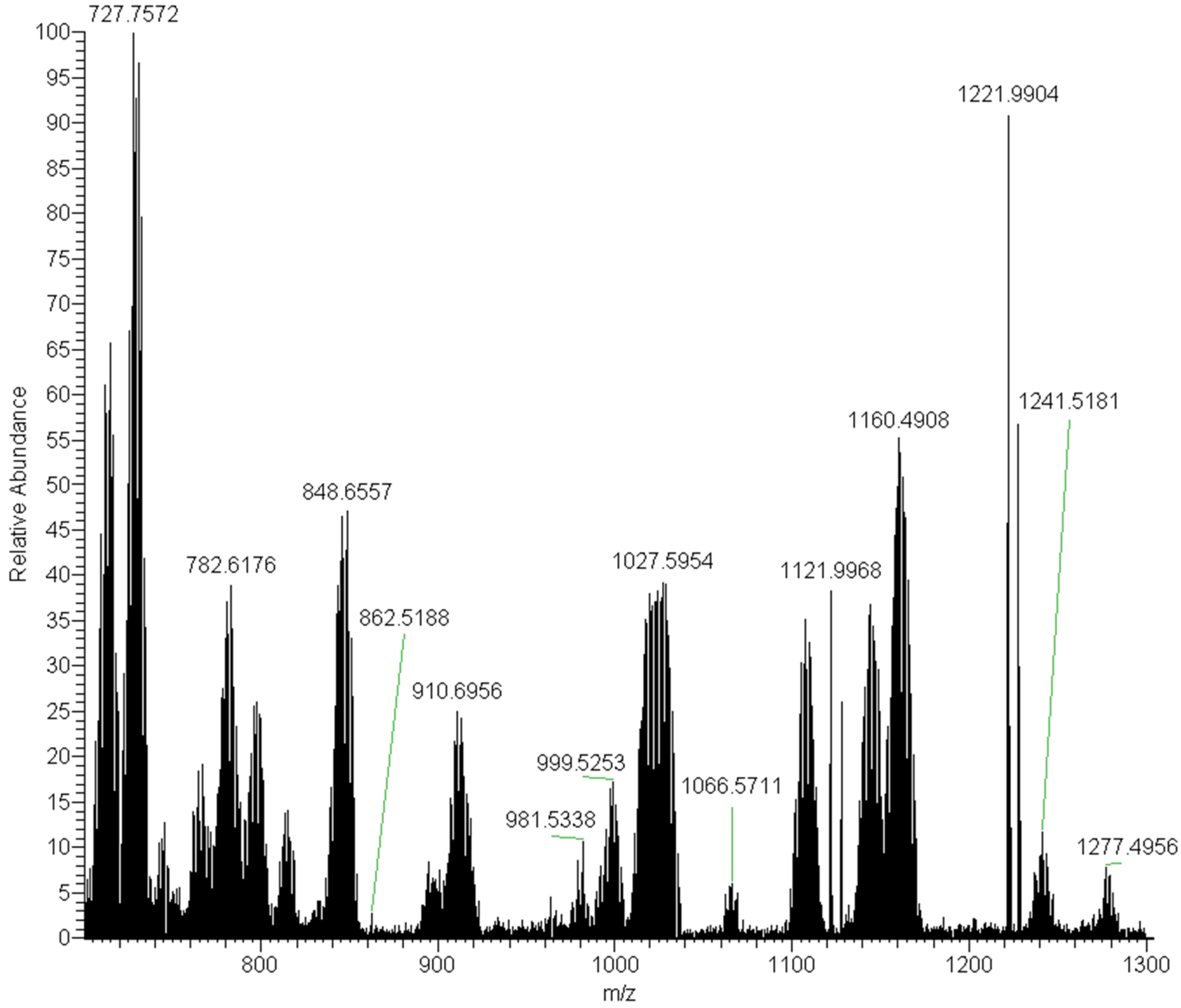

FIGURE S11. HR-ESI-MS spectrum of (**4**).
